# Test-Retest Reliability of Isokinetic Strength Measurements in Lower Limbs in Elderly

**DOI:** 10.3390/biology11060802

**Published:** 2022-05-24

**Authors:** Jose A. Parraca, José Carmelo Adsuar, Francisco Javier Domínguez-Muñoz, Sabina Barrios-Fernandez, Pablo Tomas-Carus

**Affiliations:** 1 Departamento de Desporto e Saúde, Escola de Saúde e Desenvolvimento Humano, Universidade de Évora, 7004-516 Évora, Portugal; jparraca@uevora.pt (J.A.P.); ptc@uevora.pt (P.T.-C.); 2 Comprehensive Health Research Centre (CHRC), University of Évora, 7004-516 Évora, Portugal; 3 Promoting a Healthy Society (PHeSo), Faculty of Sport Science, University of Extremadura, 10003 Cáceres, Spain; 4 Physical Activity and Quality of Life Research Group (AFYCAV), Faculty of Sport Science, University of Extremadura, 10003 Cáceres, Spain; 5 Social Impact and Innovation in Health (InHEALTH), Faculty of Sports Sciences, University of Extremadura, 10003 Cáceres, Spain; sabinabarrios@unex.es

**Keywords:** elderly, reliability, test-retest, isokinetic dynamometer, knee extension-flexion

## Abstract

**Simple Summary:**

Strength is a critical factor for maintaining the quality of life of elderly. The gold standard is isokinetic dynamometry. Thus, this study intended to measure intra-session reliability in lower limbs. The results provide excellent values and data on what can be considered a real change after an intervention.

**Abstract:**

Strength is essential for carrying out the usual activities of daily life. As there is a loss of strength in elderly, many treatments are based on delaying the loss of strength or maintaining it. Isokinetic dynamometry is the gold standard for assessing strength. It is essential that studies are conducted to allow us to identify the reliability of isokinetic strength assessments in older people. This study aimed to test the absolute and relative intra-session reliability of peak torque and work of a concentric knee extension-flexion performed at 60°/s in elderly. Fifty-two elderly subjects performed three repetitions of bilateral concentric knee extension-flexion at 60°/s using an isokinetic dynamometer. The relative and absolute reliability were calculated between the second and third repetition. The intra-class correlation coefficient values were between 0.94 and 0.98 for peak torque and work in all measures, which is considered “excellent”, except for left leg flexors in women, with values between 0.85 and 0.88, which is considered “good”. The standard error of measurement (SEM) percentage oscillated from 3.9% to 10.5%, with a smallest real difference (SRD) percentage of 10.9% to 29.2% for peak torque. The relative reliability of peak torque and work were excellent for all measures except flexors in women, evidencing the utility of isokinetic dynamometry for monitoring lower limb maximal muscle strength and work of concentric knee extension-flexion at 60°/s/s in the elderly. In addition, an SRD > 19.9% in peak torque and an SRD > 23.1% in work is considered a true change.

## 1. Introduction

Ageing is a life process that involves a series of changes, both in the body composition of the human body and in the decrease in muscle strength [1,2,3]. Muscle weakness is considered a risk factor for high mortality in the elderly [4,5,6,7,8]. In addition, ageing leads to loss of bone mass [1,2,3,4] and muscle mass [5,6,8], and reduced physical capacity [7]. Several studies have investigated changes in muscle mass and strength year by year, showing a loss of 1–2% per year in lean mass in the legs [9,10] and a strength loss of 1.5–5% per year in people older than 50 years [11]; it has even been shown that the loss of muscle strength occurs faster concerning the concomitant loss of muscle mass [10].

After the age of 75, there is a loss of strength in the lower extremities of approximately 2.5–4% per year due to different factors, such as disuse of the muscles, illness, or malnutrition [12]. This loss of lower limb strength can cause older people to have low levels of functional status and mobility [13], and low functional status, along with health problems, is associated with the risk of falls [14]. In addition, muscle strength is a determinant of independence in elderly due to its influence on physical functioning, which can lead to an inability to perform the activities of daily living or a loss of functional independence [15].

Isokinetic dynamometers are computerized instruments used for force assessment [16]. For more than 40 years, isokinetic dynamometry has been considered the gold standard for strength assessment [17,18,19]. Tests performed with this type of dynamometer allow us to evaluate maximal strength at a constant speed. The commonly used parameters are peak torque [20] and work [21]. These parameters have been used in different investigations to detect strength deficits and evaluate the effect of interventions or treatments on muscle strength [18,22]. In recent years, isokinetic tests have become the reference method for assessing strength in older adults [19]. Previous studies have used isokinetic dynamometry to evaluate knee strength in healthy older people with or without pathology [23,24]. As previously mentioned, strength in older people decreases and, therefore, it is interesting to evaluate how ageing affects this parameter [21]. With this type of tool, isometric, concentric, and eccentric strength can be tested, and in the elderly, it has been shown that isokinetic dynamometry provides reliable data during knee extension [23,24].

Knowing the reliability of assessment instruments is essential. In this sense, statistics have been developed to determine the relative reliability of an instrument, such as the intra-class correlation coefficient (ICC), and formulas have been developed to determine the absolute reliability, such as the standard error of measurement (SEM) and the smallest real difference (SRD). No intra-session test-retest reliability study has been performed with isokinetic knee flexion-extension isokinetic dynamometry at 60°/s in older adults. This study aimed to test the absolute and relative intra-session reliability of peak torque and work of a concentric knee extension-flexion performed at 60°/s in the elderly.

## 2. Materials and Methods

### 2.1. Participants

A sample size of 25 participants with 2 observations per subject achieves an 85% power to detect an intra-class correlation of 0.91 under the alternative hypothesis when the intra-class correlation under the null hypothesis is 0.75 [25] using an F-test with a significance level of 0.05. This study included 52 older people (27 females and 25 males) based on the eligibility criteria. Participants of both sexes were included if they were ≥65 years of age and signed the informed consent form. Participants with an artificial joint or severe disability that would make it impossible or contraindicated performing the isokinetic test were omitted. All participants included in the study met the inclusion criteria after reviewing their medical history.

The University of Evora Ethics Committee for research in the areas of human health and well-being approved the study protocol (reference number 16-012), which included the test reported in the present manuscript following the updates of the Declaration of Helsinki amended by the 64th World Medical Association General Assembly (Fortaleza, Brazil, 2013).

### 2.2. Measurements

#### 2.2.1. Participant Characterization Measures

An initial questionnaire was administered that asked participants’ ages, number of diseases, and number of drugs. Participants’ body weight (kilograms) and height (meters) were measured without shoes. Weight was measured with a calibrated device (Seca 760, Hamburg, Germany) and height was also measured (Seca 206, Hamburg, Germany). Body mass index (BMI) was calculated according to the following formula: BMI = weight (kilograms)/height^2^ (meters^2^).

#### 2.2.2. Isokinetic Measure

The measurement protocol followed the following sequence: firstly, a general warm-up of 5 min was performed on a cycloergometer at 100 watts and 60 revolutions per minute. Secondly, participants completed a specific warm-up consisting of 3 extension-flexion repetitions without load on each once seated on the isokinetic dynamometer according to the placement protocol. Finally, the isokinetic placement protocol was performed. Each participant was attached to the seat of the dynamometer so that the axis of their knee coincided with the axis of the dynamometer. The dynamometer remained untilted (0°), the seat orientation and isokinetic dynamometer orientation were 90°, and the range of motion and seat tilt was 85°. The weight of the testing leg was evaluated using the original Biodex System-3 isokinetic dynamometer software (Biodex, Shirley, NY, USA), and gravity adjustments were also performed using the same software.

Isokinetic test: First, 3 repetitions at 60°/s of concentric extension and concentric flexion were performed for both legs. The first repetition was considered a rehearsal for the participant to become familiar with the technical execution of the test. Repetitions two and three were used as a test and retest, respectively.

#### 2.2.3. Isokinetic Variables Studied

The variables studied were peak torque and work. Peak torque is expressed in Newton meter (N·m) and work in Joules (J). Peak torque is defined as “the single highest torque output recorded throughout the range of motion of each repetition” [20]. Work is defined as “the output of mechanical energy” [21].

### 2.3. Data Analysis

Statistical analysis was conducted using the Statistical Package for the Social Sciences (SPSS, Version 25, IBM SPSS, Armonk, NY, USA) software. The data distribution of the different variables was checked using the Kolmogorov–Smirnov test for the general sample and the Shapiro–Wilk test. The mean and standard deviation of each variable were calculated to characterize the sample.

The mean and standard deviation of peak torque and work were calculated for the test and retest. A paired-samples t-test was performed to determine statistically significant differences between the test and retest. Significance was set at *p* < 0.05. Relative reliability was calculated using the ICC [26]. The data on the chosen ICC were reported following the recommendations given in the scientific literature [26]. ICC data were calculated using the following parameters: (1) model: two-way random effects; (2) type: single rater; and (3) definition: consistency. ICC with a value above 0.90 was considered excellent, and ICC with a value between 0.75 and 0.90 was considered good [27]. Absolute reliability was calculated through the SEM and SRD [28]. The SEM was calculated with the following formula: SEM = SD × √ 1 − ICC, where SD is the mean SD of the 2 repetitions. The SRD formula was SRD = 1.96·SEM × √ 2. The SEM and SRD were expressed as a percentage. The SEM% was calculated with the formula: SEM% = SD × √ 1 − ICC/Meantest1&test2 × 100 and SRD% = 1.96·SEM × √ 2/Meantest1&test2 × 100.

To identify the level of agreement between the test and retest, a Bland–Altman analysis was performed for both peak torque and work. The *x*-axis indicates the mean of the test and the *y*-axis indicates the difference between the two measurements (retest-test).

## 3. Results

The characteristics of the general sample (n = 53) and the subsamples of women (n = 27) and men (n = 25) are shown in Table 1.

Table 2 shows the descriptive test and retest data for the peak torque and work variables. There were no statistically significant differences except for peak torque in the right and left leg flexors in the general sample and the male subsample. Significant differences between the test and retest in the leg extensors were only observed in the right leg extensors in the male subsample.

Table 3 shows relative reliability (ICC) and absolute reliability (SEM and SRD) of peak torque and work. ICC peak torque and work were excellent for all measures except for women’s flexors, which were good.

**Figure 1:**
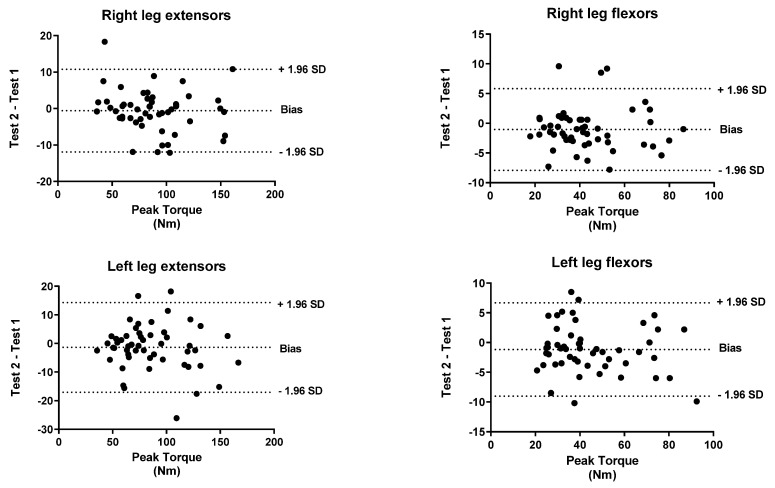
General Bland–Altman peak torque analysis for Right and Left Legs in flexion and Extension.

**Figure 2:**
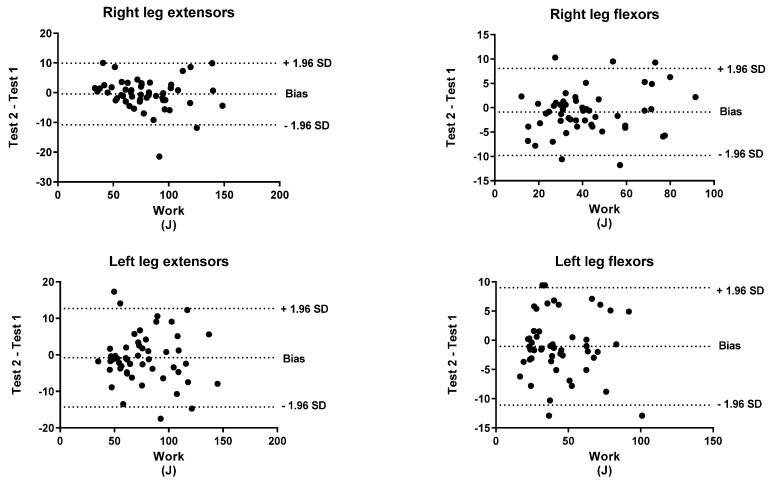
General Bland–Altman work analysis for Right and Left Legs in flexion and Extension.

**Figure 3:**
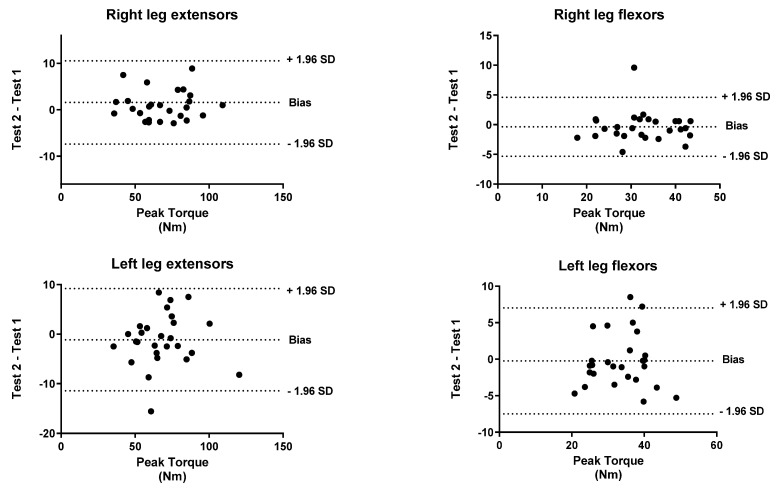
Women Bland–Altman peak torque analysis for Right and Left Legs in flexion and Extension.

**Figure 4:**
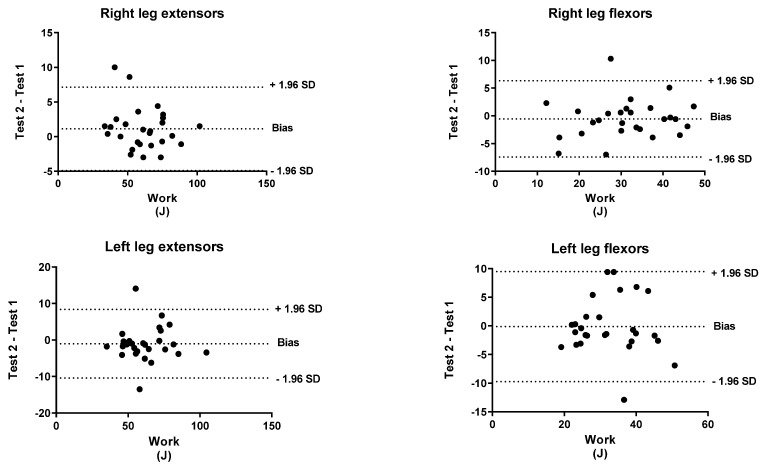
Women Bland–Altman work analysis for Right and Left Legs in flexion and Extension.

**Figure 5:**
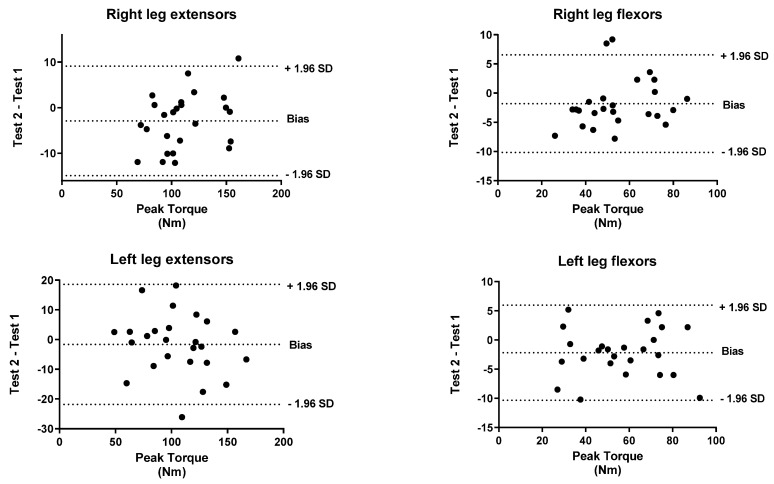
Men Bland–Altman peak torque analysis for Right and Left Legs in flexion and Extension.

**Figure 6:**
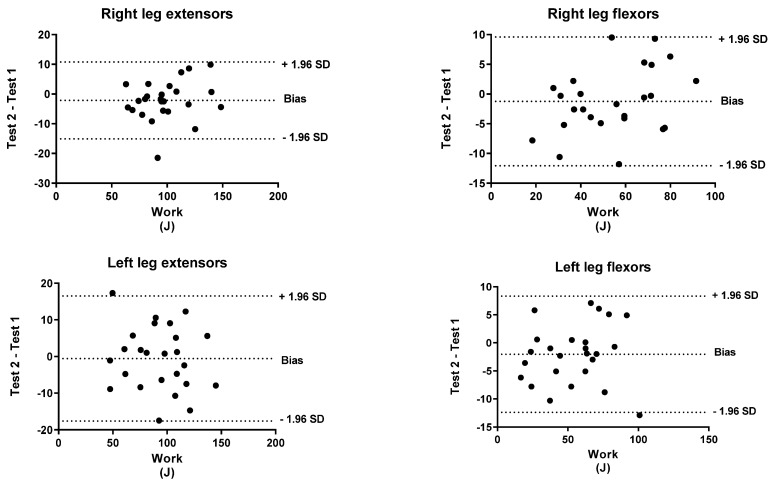
Men Bland–Altman work analysis for Right and Left Legs in flexion and Extension.

Figure 1, Figure 2, Figure 3, Figure 4, Figure 5 and Figure 6 show the general, women, and men Bland–Altman peak torque and work analysis for the right and left legs in flexion and extension.

## 4. Discussion

The results obtained from this study provide reliable measures that can be used to detect changes that are considered clinically significant in strength at low speed in response to exercise programs performed by older people. The test-retest reliability of knee extension-flexion in concentric-concentric action showed excellent reliability for peak torque and work in all measures except for left leg flexors in women (good), evidencing the utility of isokinetic dynamometry for monitoring lower limb maximal muscle strength and work in older people. On the one hand, the SRD in the case of the peak torque was between 14.3% and 19.9% in the general sample, 13.8% and 21.1% in the sample of women, and 12.0% and 18.5% in the sample of men. On the other hand, the SRD in work was between 13.7% and 23.1% in the general sample, 10.9% and 29.2% in the sample of women, and 13.3% and 22.6% in the sample of men.

Previous studies have tested the reliability of isokinetic lower limb strength measures in older people. In this sense, studies including participants between 65 and 82 years old, similar to the present study, but assessing knee extensor strength at a knee joint angle of 90°/s, showed good to excellent reliability, with values ICC = 0.85–0.98, SEM: 3–10% [29], good to excellent reliability (ICC = 0.84–0.94) [30], excellent reliability (ICC = 0.93) [31], and good to excellent reliability values (ICC = 0.81–0.99) for isokinetic knee and ankle contractions and most SEM indices, representing an acceptable concordance from 6 to 13 [32]. Moreover, several studies have been carried out in older people with different pathologies and disorders. A trial conducted with type 2 diabetes mellitus, following a protocol of three repetitions of extension-flexion in concentric action at 60°/s on both legs, found excellent reliability for peak torque and work. The SEM ranged from 3.85% to 6.80%, and the SRD from 10.66% to 18.86% for peak torque, finding that an SRD < 20% was indicative of a true change after the intervention [33]. Chronic obstructive pulmonary disease patients were also studied [34,35,36,37]. These patients performed 30 maximal knee extensions at angular velocities of 90 and 180°/s in random order, showing that the reliability at 90°/s was excellent at 0.97 (0.92–0.99) and an SEM percentage of 12 for peak torque, with this being the most reliable option. Isokinetic in the lower limbs was tested in 257 sarcopenic and malnourished older adults (men = 98 aged 76.8 ± 6.3 years and 159 women aged 75.9 ± 6.6 years). They completed, among other measures, a lower limb isokinetic test at different times. At baseline, men obtained ICC = 0.93, SEM = 7.12, and SRD = 19.72, and women ICC = 0.91, SEM = 4.70, and SRD = 13.02 [26].

In the present study, the peak torque reliability of men and women was similar in all studied actions except in left leg flexors, which were slightly lower in women (ICC < 0.90; %SRD > 20%) versus men (ICC > 0.95; %SRD = 16.5%). As shown in the Bland–Altman graphs for peak torque and work for the entire sample, the BIAS is negative. This means that the values of the retests were lower than those of the test (given that the Bland–Altman was calculated by subtracting the retest minus the test). However, this BIAS is around 0.2 and 0.5 1 N·M at peak torque and around 1 and 2 J at work. This means that this bias is very small since it does not represent more than 2% of the mean values obtained, so we believe that it is a low and acceptable bias.

The practical implications of this study are (1) peak torque and work can be used as reliable parameters for monitoring strength in older adults over 65 years; (2) both peak torque and work can be used to measure changes in these parameters such as after an intervention; and (3) the reliability of left leg flexor strength was lower in women, which should be considered when interpreting changes, such as after an intervention, increasing the SRD.

Isokinetic dynamometry has several advantages and disadvantages compared to other traditional methods and tests. In general, isokinetic dynamometry has the disadvantage that it is not easily adaptable to different contexts (it is difficult to use in common contexts such as nursing homes, patients’ home, etc.) and is expensive compared to other traditional tests. This often means that it is relegated to clinical studies with adequate funding or clinical practice for pathologies that can benefit from it. As a great advantage, it is considered the gold standard. On the other hand, isokinetic force measurement is the closest technique to capturing a real, physiological muscle contraction [38] and, in general, has excellent reliability in most populations in which it has been studied, as is the case in this study.

There are a number of traditional tests that are highly reliable and have the great advantage of being easily and quickly used by researchers. The chair test is one of these tests, which can be used to assess rehabilitation [39], functional fitness level [40], and monitor training [41,42]. With this test, the state of physical performance is known, with low (30 s-CST ≤ 8 repetitions) to high (30 s-CST > 8 rep) results [43]. The minimum detectable change is 1.6 repetitions in patients with osteoarthritis [44] and 3 repetitions in patients with Parkinson’s. Another test is the five-repetition sit-to-stand test. This test has good reliability in older adults [9] and the minimum detectable change time (MCD) for the test is between 3.6 and 4.2 s [10] while the minimum clinically important difference (MCID) is 2.3 s [11]. However, these tests have a number of disadvantages, including that they cannot be performed on subjects suffering from mobility limitations. Comparing both tests, the 5-chair STS test is an indirect indicator of lower extremity speed and muscle power while the 30-s STS test is an indirect indicator of lower extremity endurance (muscle capacity). Therefore, both tests are complementary. Another test is the 10 repetitions of sit-to-stand, where the minimum real change is 9–10% [45]. Finally, another test is the leg press/leg extension test. This type of test is used to quantify the 1 RM (1 repetition maximum resistance) of leg strength, where the evaluation is performed with the highest resistance for which the subject can complete the exercise only once. The minimum detectable change of this test is 0.4 kg per 1-RM leg press, which means detectable changes of 1.1% [46]. This test has excellent relative reliability (ICC > 0.94) among community-dwelling older adults [14]. However, the disadvantage is that these resistance machines do not require activation or engagement of any of the major stabilizer muscles and is a widely used exercise to strengthen the lower limbs. This test is performed using closed-chain kinetic effort [15], whereas knee extension involves large muscle groups of the lower body (i.e., quadriceps and hamstrings) [47].

This study has several limitations. All the participants were community-dwelling older people between 65 and 86 years old, so the interpretation of the results might not apply to those over 86 years old, clinical populations, or frail elderly. Another limitation was related to the measures bias: (a) the alignment of the mechanical axis of the isokinetic dynamometer to the knee was performed manually with a visual inspection, with the possibility of influencing the minimal difference in the placement between the test and retest, and (b) difficulties in determining whether the participants performed each repetition with the maximal effort. Even though all participants were verbally encouraged to perform maximal strength from the beginning to the end of the test, no standardized recording of this verbal incentive was made, contributing to differences between stimuli. Lastly, as only one angular velocity (60°/s) and muscular action (concentric) were measured, how the test would behave at other angular speeds and muscular movements is unknown.

## 5. Conclusions

Isokinetic concentric knee extension-flexion performed at 60°/s demonstrated excellent reliability for peak torque and work measurements, except for flexors in women, which was good. In addition, our results showed that, in general, the smallest real difference of 20% in peak torque and 24% in work can be considered a true change in strength regarding knee extensor and flexor muscles at 60°/s, which will assist therapists and clinicians in interpreting assessments of isokinetic knee extension and flexion tests in the elderly during clinical practice.

## Figures and Tables

**Table 1:** Sociodemographic characteristics.

	Total Mean (SD)	Women Mean (SD)	Men Mean (SD)
Age (years)	73.25 (4.92)	74.56 (5.36)	71.84 (4.04)
Height (m)	1.59 (0.09)	1.52 (0.06)	1.66 (0.07)
Weight (kg)	77.42 (20.82)	67.65 (10.67)	87.96 (23.98)
BMI (kg/m^2^)	30.33 (7.45)	29.02 (4.55)	31.73 (9.56)
Diseases (n)	3.46 (1.55)	4.05 (1.47)	2.40 (1.07)
Medication (n)	3.93 (2.63)	4.94 (2.41)	2.10 (2.02)

SD: Standard Deviation; BMI: Body Mass Index; n: number.

**Table 2:** Summary of Isokinetic Peak Torque and Work.

Peak Torque (N·m)						Work (J)				
	Test		Re-Test			Test		Re-Test		
Test Measurement	Mean	(SD)	Mean	(SD)	*p*	Mean	(SD)	Mean	(SD)	*p*
General										
Right leg extensors	88.65	(32.68)	88.08	(31.74)	0.480	79.42	(28.06)	78.97	(27.30)	0.545
Left leg extensors	86.75	(32.88)	85.38	(30.82)	0.223	77.36	(26.92)	76.58	(26.45)	0.414
Right leg flexors	43.78	(16.76)	42.73	(16.68)	0.035	42.69	(18.68)	41.81	(19.57)	0.171
Left leg flexors	45.19	(18.73)	44.02	(18.18)	0.040	43.61	(20.85)	42.56	(20.60)	0.149
Women										
Right leg extensors	66.66	(19.76)	68.24	(19.03)	0.083	60.69	(17.29)	61.82	(16.67)	0.067
Left leg extensors	68.82	(18.04)	67.70	(18.26)	0.277	62.79	(15.23)	61.77	(15.39)	0.280
Right leg flexors	32.69	(7.49)	32.33	(7.47)	0.462	31.54	(9.62)	30.99	(10.13)	0.428
Left leg flexors	33.64	(7.31)	33.41	(7.44)	0.742	32.59	(8.94)	32.47	(8.74)	0.898
Men										
Right leg extensors	112.41	(26.73)	109.51	(28.76)	0.026	99.66	(22.97)	97.50	(24.39)	0.116
Left leg extensors	106.11	(32.49)	104.48	(30.43)	0.436	93.10	(28.14)	92.57	(26.79)	0.781
Right leg flexors	55.75	(15.77)	53.96	(16.67)	0.046	54.73	(18.72)	53.49	(20.71)	0.273
Left leg flexors	57.66	(19.39)	55.48	(19.46)	0.015	55.50	(23.53)	53.47	(24.09)	0.066

N.m: Newton meter; J: joules; SD: Standard Deviation; *p*-value of the Paired-Samples *t*-Test.

**Table 3:** Absolute and relative test-retest reliability of the Peak torque and Work.

Assessed Action	ICC (95% CI)	SEM (N·m)	SEM (%)	SRD (N·m)	SRD (%)	ICC (95% CI)	SEM (J)	SEM (%)	SRD (J)	SRD (%)
Total (n = 52)	Peak Torque (N·m)					Work (J)				
Right leg extensors	0.98 (0.97–0.99)	4.56	5.20	12.63	14.30	0.98 (0.97–0.99)	3.91	4.90	10.85	13.70
Left leg extensors	0.97 (0.94–0.98)	5.52	6.40	15.29	17.80	0.97 (0.94–0.98)	4.62	6.00	12.81	16.60
Right leg flexors	0.98 (0.96–0.99)	2.36	5.50	6.55	15.20	0.97 (0.95–0.98)	3.31	7.80	9.18	21.70
Left leg flexors	0.97 (0.96–0.98)	3.20	7.20	8.86	19.90	0.97 (0.95–0.98)	3.59	8.30	9.95	23.10
Women (n = 27)										
Right leg extensors	0.97 (0.94–0.99)	3.36	5.00	9.31	13.80	0.98 (0.96–0.99)	2.40	3.90	6.66	10.90
Left leg extensors	0.96 (0.91–0.98)	3.63	5.30	10.06	14.70	0.95 (0.90–0.98)	3.42	5.50	9.49	15.20
Right leg flexors	0.94 (0.88–0.97)	1.83	5.60	5.08	15.60	0.94 (0.87–0.97)	2.42	7.70	6.70	21.40
Left leg flexors	0.88 (0.75–0.94)	2.55	7.60	7.08	21.10	0.85 (0.70–0.93)	3.42	10.50	9.49	29.20
Men (n = 25)										
Right leg extensors	0.97 (0.94–0.99)	4.81	4.30	13.32	12.00	0.96 (0.91–0.88)	4.74	4.80	13.13	13.30
Left leg extensors	0.95 (0.88–0.98)	7.03	6.70	19.50	18.50	0.95 (0.89–0.98)	6.14	6.60	17.02	18.30
Right leg flexors	0.96 (0.91–0.98)	3.24	5.90	8.99	16.40	0.96 (0.91–0.98)	4.41	8.10	12.22	22.60
Left leg flexors	0.97 (0.94–0.99)	3.36	5.90	9.33	16.50	0.97 (0.94–0.99)	4.12	7.60	11.43	21.00

N.m: Newton meter; J: joules; ICC: Intraclass Correlation Coefficient; CI: Confidence Interval; SEM: Standard Error Measurement SRD: Small Real Difference.

## Data Availability

Datasets will be available under reasonable request.
